# Collectanea

**Published:** 1829-02

**Authors:** 


### COLLECTANEA.

Floriferis ut apes in saltibus omnia libant,
Omnia nos, itidem, depascimur aurea dicta.

### ANATOMY.

*Conformation of the Human Stomach.*—From some recent observations of S. Th. Soemmering on this organ, it appears,

1. That the Negro stomach differs from the European in being more round, and more nearly resembling that of the monkey. This rotundity is particularly evident at its great curvature.

2. The contraction which is occasionally seen in the middle of the stomachs of some subjects, is found almost exclusively in those of females, and is apparently produced by the constant and excessive pressure of their stays upon the epigastrium. No trace of this malformation is found in children.

3. Finally, the pyloric outlet is different in various individuals, and the modifications of this orifice, (which the author, from his own observations, reduces to four classes,) are chiefly produced by a glandular zone, of rather a hard consistence, which forms the outline of the opening, and which may be discovered by cautiously elevating the peritoneum and subjacent cellular membrane.—*Denkschrif., desk Akad. d. Wissensch. Z. München*, tom. viii.

### PHYSIOLOGY.

*Remarkable Case of Preternatural Fœtation.*—The following singular case was published many years ago, by Dr. J. M. Good, in a little work which may probably not have fallen into the hands of our readers.

This case of preternatural fœtation was one of twins, the first of which was born alive. It had no sexual characteristic, neither penis nor pudendum. It had neither anus, funis, nor umbilicus. The legs were distorted and curved outwardly. It cried feebly once or twice after birth, and died in about ten minutes. With a little force, a small, empty, and shrivelled placenta followed, in which neither funis, umbilical vessels, nor any other appendage by which it could have been attached to the child, could be traced. No blood followed its removal from the uterus. The other child was perfect in every respect. On dissection, it was found that the right kidney adhered to, and communicated with, the bladder. There was no urethra, nor internal organs of generation; no anus, nor rectum, the colon terminating insensibly in the peritoneum.

This singular case naturally led Dr. Good into some reflections as to the mode in which the fœtus receives its nourishment in the womb, whether from the mother, through the medium of the placenta and umbilical vessels, or by the liquor amnii? He deems this case conclusive against the exclusive power of the placenta. His opinion is, that the placenta is the proper organ of oxygenation for the fœtus, and the liquor amnii its proper source of nourishment. The former, he conceives, is capable of communicating nutriment whenever the amnios may fail, or be suddenly discharged; and that the amnios is, on the contrary, capable of communicating oxygen whenever the placenta is either defective or wanting.

---

### PATHOLOGY.

*Diseases of the Spleen.*—It is commonly said that, the spleen being subservient to the liver, the diseases of these two viscera are similar, and should be treated in the same manner. But there never was a more fallacious assumption than that which imagines a similarity of diseases of these two organs, nor a more false conclusion than that which suggests the employment of corresponding treatment for their respective diseases.

In the structure of the liver, the chief points of difference are its firmer and less distensile substance, the absence of an elastic cellular structure connected and freely communicating with the branches of the blood-vessels, besides the existence of an excretory duct; while the spleen appears in every point of view fitted for, and subservient to, functions totally different from secretion.

Resulting from the structure of the spleen just stated, we see it performing a train of functions as different in their nature from those of the liver as could possibly be anticipated from considering organization so totally dissimilar.

The best authenticated observations concerning the spleen tend to establish the constancy of the following phenomena:

1. When a person is suffering under the cold fit of an ague, or when by any cause the blood is driven from the surface to the interior of the body, there is general congestion of internal organs; but at the same time the spleen enlarges, and, receiving an increased load of blood, saves other parts of different structure from distention and disease.

2. On descending into the cold bath, the muscles of respiration act spasmodically; at the same time that the blood, driven from the surface by cold, causes a turgid state of the spleen, which forces up the diaphragm. A child, therefore, when put into the cold bath, will say it takes away his breath. The unpleasant sensation is felt most at the left side, and arises in consequence of the diaphragm being pushed up by the spleen, and thereby encroaching on the left side of the chest and heart.

3. When, from riding, running, or other violent exertion, the circulation is subject to sudden agitation, the spleen becomes distended and painful, from accumulation of blood. Long-continued and violent laughter causes a sensation as if the side were splitting. But exercise, gradually increased until there be a free perspiration, is very rarely attended with pain in the left side.

4. When the system is suffering from the tumultuous agitation of ungovernable anger, nature would be overpowered, and the vessels of some weak part would burst, were not the distensile structure of the spleen ready to admit a temporary accumulation of blood, and thus preserve other organs from injury.

Under all these circumstances of circulation suddenly repressed from the surface of the body ; as in the cold stage of intermittent fevers, or by cold applied to the skin ; also in cases of sudden agitation of circulation, by riding, running, or violent laughing, or during the tumultuous movement of violent passions of the mind ; we find the spleen acting the part of a safety-valve to obviate sudden and violent pressure,* under which the vascular system would otherwise in some parts be apt to burst. In these cases, Dr. Rush truly says, "the spleen performs the office of a basin held by the hand of nature, to receive for a while several pounds of blood, in order to preserve the system from disease and death."

The experiments of Magendie show the effect which changes in the balance of the circulation produce on the size of the spleen. He states, that if the abdomen of a living dog be opened, so that the spleen and other viscera can be observed, and a pint of blood injected into one of the veins of the extremities of the animal, the spleen will increase one-third or one-half beyond its former dimensions, and be infinitely more distended than any other internal organ. Also, if the abdomen of a dog be opened, and the viscera viewed while the animal is bled to fainting, the proportional decrease in the size of the spleen is much more than the decrease of other viscera.

The fact that men have, in a few rare cases, survived the loss of a portion of the spleen, and in one or two instances even of the whole of that viscus, can hardly be urged against the facts and opinions above stated, until repeated observations of similar cases in man shall prove that the absence of the spleen can be generally endured without injury, and that the action of no other organs can compensate for its loss.

Even in the slow and silent operation of long-continued grief and distress of mind, whereby the secretions are perverted, the cutaneous circulation becomes languid, transpiration is obstructed, and we often find enlargement of the spleen takes place. Such cases are always difficult to cure.

My observations on the uses of the spleen entirely concur with the opinions of Dr. Benjamin Rush. Sentiments somewhat similar to those of Dr. Rush

* Repeated overdistention of the spleen is not endured with impunity by patients who have intermittent fevers, especially if they have been long subject to the debilitating influence of miasmata. After a person has been subjected to frequent returns of ague, or of remittent fever, the spleen is not only large, but there is morbid sensibility on pressure. Repeated overdistention during febrile paroxysms gives rise to a sort of internal subacute inflammation; in which state a free use of stimuli is followed by that form of disease which I have denominated the irritative stage of tumid spleen, and its consequence is frequently a thickening of the internal structure, by which the contractile power of the spleen is diminished or lost; and, unless remedied by timely means, an intractable chronic induration of the spleen is the result.

When the spleen has become diseased and incapable of distention, hemorrhages from the nose, lungs, or stomach occur. If profuse, these hemorrhages suddenly destroy life; but, when more moderate, they almost always afford temporary relief, and if the other functions of the system (that have been disordered) be then restored, the patients sometimes recover entirely from the local disease of the spleen.

are adopted by Mr. Hodgkin, who has written an ingenious essay on this subject.—*Extract from* Mr. Twining's *Paper on Diseases of the Spleen, in the Transactions of the Medical Society of Calcutta.* 171

---

*Case of Hydrophobia; by* Mr. F. Godrich.—On Thursday morning, the 25th November, I was called up, about seven o'clock, to see a man who I understood was exceedingly ill, and waiting in the surgery very impatiently for my arrival. I found my patient (Mr. Barham), a fine looking old man, about sixty, labouring, at intervals of about five minutes, under strong spasmodic paroxysms, affecting the muscles concerned in breathing and deglutition. There was a wildness and an impatience depicted in his countenance, totally different from any thing I had ever observed in other spasmodic affections. His bowels were open, tongue clean, skin moist, pulse full and a little accelerated. I took away twenty ounces of blood, and prescribed a mixture containing ʒss. of laudanum for a dose every hour, until I should see him again. He walked home, half a mile from my house (Gloucester road, Old Brompton), and left me ruminating on the probable cause of so much mischief occurring suddenly in a fine healthy subject.

At ten, a message was sent, saying that he was much worse, and requesting me to call as soon as possible. He received me tranquilly, and said he was very glad that I had come to see him, for he was very ill. His symptoms were now more distressing than when I first saw him: he looked wildly and suspiciously at every one entering his apartment, and his breathing was accompanied by a short convulsive sobbing. On looking at his medicine, I perceived he had taken none, and expressing my surprise, he assured me it was impossible for him to swallow a single drop, as the attempt had been followed by violent spasms, and produced so much distress that he had desisted. At this period no one had the slightest idea of the origin of his malady. I poured out some medicine into a teacup, the very act of which produced much excitement and alarm. My first impression as to the true nature of his disease arose at this period, from the circumstance of his requiring a teaspoon, with which he endeavoured to take some of the medicine. The attempt produced much excitement and alarm, and after two or three painful efforts at deglutition, with one desperate effort he swallowed a teaspoonful, threw away the spoon, and begged, unless I wished to destroy him, that he might have nothing more to swallow. I now left the room, and inquired of a bystander whether any thing particular had occurred to him within the last few weeks? On recollection she said, "About a month since, late at night, a strange dog came into the premises, and fought with his own dog; he got out of bed to separate them, and the strange dog bit him in two places, on the left arm and hand; and bit a puppy, which died about a fortnight after in a strange way, which was thought to be some kind of a fit." To ascertain if this occurrence had produced any effect on his mind, while again bleeding him I said, "You have been in the wars, sir, and had your hand and arm torn: how did it occur?" "Oh!" said he, carelessly, "that was done by a dog a long time ago, but it healed." The circumstance was never again mentioned to him, and he died in total ignorance of the cause of his malady. The wounds were perfectly cicatrised, and there was not the least action going on indicative of recent absorption. He bore the bleeding pretty quietly: forty ounces were removed, which on cooling presented strong marks of inflammation.

Ordered ♏iv. Acid. Hydrocyan. omni horâ, in a little water.

Twelve o'clock.—With much difficulty he has taken two doses of the acid; pulse full and hard, 110. Thirty ounces more blood were removed.

Three o'clock.—Has taken two more doses. Complains of a dreadful sense of suffocation, and implores that nothing more may be given him. Pulse full, and beating at 120 to 130.—Continue the acid.

Eight o'clock.—Pulse full and hard. Has taken in all 24♏. of the acid; but so painfully distressing has the deglutition now become, that all attempts at repeating his medicine are discontinued.—V.S. ad ℥xxx.

During the bleeding, he looked wildly at the basin, and begged that no more might be spilt, (a drop or two had fallen,) repeating frequently, in great agitation, as the blood was running, "Take care! take care!"

Between two and three o'clock next morning, my assistant (Mr. Davies) visited him. He found him tolerably passive, but observing every movement with intense anxiety. Pulse full and hard, face flushed, eyes denoting cerebral irritation. He had been at times outrageous. On its being intimated that bleeding was again necessary, a paroxysm came on more intense than any preceding, and with great effort he submitted. As the blood flowed he became more and more alarmed, till at length he got quite unmanageable: he raged violently at his nephew, who was holding the basin, and ordered it peremptorily to be removed. Thirty or forty ounces were taken away. It was found necessary to put on the strait-waistcoat.

About four o'clock Mr. Davies wished him to take some more of his medicine. He said "I can take no more;" and, on reaching the bottle to put out a few drops, he became violently agitated, threw himself from side to side, and, as well as the incessant spasmodic sobbings would allow, he begged that not one more drop of any thing might be offered him, and that the bottle might be taken from his sight. He did not become tranquillised until its removal.

He lingered on till ten A.M. in the same state, a few minutes before which he insisted on getting up, and walked a short way down his garden, returned, laid down on his bed, and died.

Mr. Frederick Salmon, of Old Broad street, and Mr. Wilson, of Chelsea, were kind enough to assist me in conducting the post-mortem examination. On opening the chest, the heart was free from disease, with rather more water in the pericardium than natural. The lungs were completely gorged with grumous blood, and the pleura adherent on the right side. On removing the cranium, which was remarkably thin, and cutting the substance of the brain, numerous red spots presented themselves in the medullary portion; about a tablespoonful of water in each ventricle; the plexus choroides was turgid; the corpora striata, thalami, and basis of the brain, every where preternaturally injected; the cerebellum, crura cerebri, and cerebelli, in a high state of inflammation. On removing the spinous process of the vertebræ, the whole cord was considerably inflamed; and, opposite the two last cervical and dorsal vertebræ, the cellular substance was studded with dark patches of coagulated blood, the theca vertebralis thickened, and the cord in an active state of inflammation. The larynx and pharynx bore not the slightest vestige of disease.

The preparation of the cord is deposited in the museum of the London University.

The post-mortem examination of this case tends to prove the correctness of Professor THOMPSON's theory of the proximate cause and seat of this afflicting malady; and the plate accompanying a case recorded by him, in the 13th volume of the Medico-Chirurgical Society, gives a faithful delineation of the state in which the spinal cord was found in this case.—*Med. Gazette.*

### PRACTICAL MEDICINE.

*Acupunctuation in India.*—The Hindoo physicians, when ordinary means have failed in cases of diseased spleen, have recourse to the introduction of iron needles, heated red hot. They are plunged into the side, above the spleen, in various places, according to the discretion of the operator, usually not fewer than seven nor more than twenty. It seems likely that they penetrate its substance. The needles are made to penetrate to a considerable depth, according to the native's idea, through the spleen. The operation is attended with violent pain, but is said rarely to fail of success.

### *Acupunctuation.*

CASE I. *Hysteria, with Paralysis and Contraction of the Limbs.*—A woman, twenty-eight years of age, of a delicate constitution, whose general health was bad, and who had been from the age of fourteen subject to hysteria, and various remedies having been tried, to remove the disease, such as general and local bleedings, blisters, the use of the vapour bath, accompanied by the successive exhibition of all the known antispasmodics, without the least success; she was admitted into the Hôtel Dieu, with the following symptoms:

Emaciation of the body; countenance clay coloured; hemiplegia of the left side; tongue red, and incapable of being protruded out of the mouth; apyrexia; thirst; loss of appetite; nausea; a fixed and circumscribed pain of the epigastric region; a periodical pain in the hypogastric region, before and during the fits; hardness and tenderness of the abdomen; with loss of speech, after the fits.

By bleeding twice, and by the use of diluents, the speech was restored, and also the use of the left side. But shortly afterwards two fresh attacks of hysteria presented themselves: they were preceded by the usual symptoms, immoderate laughing; excessive irritability of the senses; violent palpitations of the heart; a universal sweat; an acute pain in the occiput and along the spine; respiration short, irregular, and frequent. The fit then showing itself by constriction of the throat, sometimes with the sensation of a ball rising from the abdomen into the chest, the head being thrown back, the eyes being wide open and fixed, the pupils dilated and insensible to the action of light, loss of hearing and smelling, foaming at the mouth, deglutition impossible, stertorous breathing, convulsive motions of the muscles; flushed and bloated countenance; hypogastric region tense, hard, and painful to the touch: all these phenomena diminishing at intervals, and during the intervals she experienced great thirst and desire to drink cold water; but no sooner had the patient swallowed some of the fluid, than all the above symptoms were renewed with their previous severity. After the space of two hours, the time which the fit lasted, she recovered her senses; the palpitations of the heart were diminished; the pain of the occiput and that of the hypogastrium continued for some hours, with great thirst, tongue red and dry, limbs benumbed.

A few days afterwards, a similar fit took place to that described above; after which there were hemiplegia of the left side, immobility of the tongue, aphonia, difficult deglutition, a frequent strong pulse. She was bled generally and locally, and antispasmodics were administered, but without any good effect.

She remained in this state for three days, when menstruation took place, which had ceased for the last ten months. In the evening of this day, the patient could articulate some words in a low voice, and the tongue was more capable of being moved.

After the lapse of a week, the contraction of the limbs still continued very strong and very painful. She was bled in the contracted arm, and, as soon as the lancet had penetrated the vein, the fingers, which were before bent upon the forearm, extended, and acquired their usual movements.

The contraction of the leg continued, with pain in the loins, for three days, notwithstanding general and local bleedings, baths, &c.; when four needles, of an inch and a half to two inches in length, were introduced into the lumbar region. Three hours after they were withdrawn, the patient could walk, to her great astonishment. The pain of her loins also was removed.

The next day she had a violent pain in the left thigh. Two needles were introduced on the posterior part of the leg, in the tract of the sciatic nerve: the pain subsided, and on the same day she was able to walk freely.

On the following day there was again a pain in the internal part of the leg and upon the foot. Two needles were introduced into the painful part of the leg, and in four hours after they were removed, when the pain almost instantaneously ceased.

Two days afterwards, two needles were placed in the back part of the foot, and after their removal the pain subsided, and the movements of the foot were free and easy.

In a week the patient was quite free from pain, and could use her limbs freely; when in the morning she began to experience the precursory symptoms of a fresh attack of hysteria. Six needles were immediately introduced: two in the cervical region, two in the dorsal, and two in the lumbar. All the symptoms disappeared, and the fit did not take place.

On the evening of the ensuing day, the same precursory symptoms manifested themselves, but less violent than those of the day before. Three needles were immediately introduced into the cervical region near the occiput, where the pain was the greatest; and all the symptoms were removed.

Five days afterwards, the patient, knowing that she was about to have a fit, desired that the needles might be introduced. Four were placed in the neighbourhood of the occiput, and the fit was completely prevented; some muscular contractions only taking place after the introduction of the needles.

Having for many days experienced no precursory symptoms of hysteria, she left the hospital with the idea that she was cured; but during the month of the following May she experienced a numbness in the left arm, the catamenia having been suppressed by fright. On the 17th of May she returned to the hospital, and experienced every day one or more hysterical fits, which brought on again hemiplegia of the left side, contraction of the limbs, and even numbness of the right hand.

The means which were employed when she first entered the hospital in January were again repeated, and presented the same phenomena, and were crowned with the same success. In fact, the hysterical fits were again dispersed by acupunctuation. The hemiplegia and the pains were removed by this means; and the bleeding removed the contraction of the arm a second time, as soon as the puncture with the lancet was made.—*Archives Générales.*

---

### SURGERY.

*Femoral Aneurism, by* M. Dupuytren. *Difficulties and Dangers anticipated.*—On November 18th, a man was brought into the amphitheatre, with an aneurism of the femoral artery, about nine inches in length, and five or six in breadth. On being questioned as to its origin, he stated that, about a month previously, he was attacked with shivering, and soon afterwards, feeling pain in the thigh, he examined the affected spot, where he discovered a small tumor. As aneurism is not commonly ushered in by shivering, he was again particularly interrogated by M. Dupuytren, and remained firm in his statements; but the man seemed to be in such a state of depression and stupidity, that no great reliance could be placed on his account.

The pains had latterly been lancinating, and shooting down into the leg. Some person, whom he had consulted, applied leeches and cataplasms of hemlock, with acetate of lead. The symptoms, and among them the shiverings, continued for a fortnight. At length he was unable to walk; and, during the space of another fortnight before his admission into the hospital, he was confined to his bed, where the tumor daily increased in size, while his general strength declined. The pulsations were manifest to the eye at a distance of ten yards.

On the morning after admission, the increase of the swelling was so great as to render the immediate application of the ligature necessary, as delay might have been speedily fatal, by the rupture of the sac. Although no doubt could be entertained of the necessity for the operation, from the imminent danger that existed, yet the circumstances of the case excited great uneasiness in the mind of M. Dupuytren; and, in giving a clinical account of the difficulties he should have to contend with, and, above all, the uncertainty of success, he eloquently yet briefly depicted the wretchedness of the surgeon under these circumstances.

It was deemed advisable to place the ligature as near as possible to the upper edge of the tumor, and this was just below the part where the profunda is usually given off. "We know (says M. D.) that if we tie a vessel near the part where a large branch is given off, the coagulum cannot form. But does this necessarily prevent the obliteration of the tube by adhesion? I think not; and were it otherwise, the ligature might be carried above: for, if the circulation of the limb can continue after the ligature on the external iliac, it would be unimpaired by the same operation in the groin." The possibility of cutting the vein was suggested, in which case M. Dupuytren proposed to divide it, and tie the two ends.

The integuments were distended with fluid, and the anatomical character of the part he considered less favorable for the application than under the sartorius. Admitting that it required more caution and skill, yet the difficulty could not be considered great. The bare notion, indeed, of mishap in the present instance arose from the rapid growth of the tumor; and the chances of failure, from the depression of the patient, and from other circumstances over which we can exercise no control.

It would be unfair to suggest that M. Dupuytren wished to load his colleague with a burthen, the weight of which he felt himself both unwilling and unable to support. Be that as it may, he offered to resign the operation to M. Sansom; which he politely declined accepting.*

The man being placed on the table, an incision was made down to the aponeurosis, which was carefully opened, and, by means of a probe-pointed and curved bistoury, divided upwards and downwards. The artery was discovered, separated from its accompanying vein and nerve, and a small ligature was applied in the usual way.

The morning after the operation, no unfavorable symptoms had occurred. He was free from pain about an hour after the operation, and had seven hours of uninterrupted sleep. He had been bled, and warm flannels had been constantly applied to the leg.

His tongue is coated, and his pulse full, but there is not the slightest reason at present to despair of his recovery. *(From a Correspondent at Paris.)*

---

*Cases of Excision of the Uterus.*—Case I.—Fany Guillon, thirty years of age, experienced, about six years ago, pains in the womb, which came on after her delivery. Her sufferings having greatly increased, she was examined, and a considerable softening of the neck of the womb, with ulceration, was discovered.

She was bled, and the vagina was filled with a liquid cataplasm and the Decoctum Solani nigri.

On the following morning, on examination by means of a speculum, the neck of the uterus was found four times its natural size, and softened to the extent of three-fourths of its surface. On the left was a hard point, at first supposed to be a tubercle. Ecchymoses were perceived on the surface, with small vegetations, which bled on the smallest contact. A deep ulcer existed round the os tincæ, in extent about two lines. Slight erosions were here and there on the other part of the cervix. These were accompanied by lancinating pains, which were intermittent.

M. Lisfranc thought that the only course to be pursued was amputation, but determined on trying the effect of the ordinary treatment. Bleeding, injection of liquid cataplasms and of decoction of solanum, warm baths, warm hip baths, emollient clysters, severe and vegetable regimen, were tried during seven weeks.

Towards the end of August, the progress of the affection was observed to be unfavorable: it threatened to usurp the whole vaginal insertion of the peritoneum. The emaciation and debility of the patient sensibly increased; yellowness of the skin was strongly marked. A fetid discharge took place, and was accompanied by acute lancinating pains. Under these circumstances it was thought advisable no longer to defer the operation.

The smallness of the projection of the womb in the vagina compelled M. Lisfranc to perform the incision within this canal. The softening of the

* M. Sansom is the second surgeon of the Hôtel Dieu, and into his ward the patient was first received. But the junior surgeons of the French hospitals never perform operations, unless through the complaisance of their seniors.

posterior part of the cervix was such that the double branch of the hooked forceps caused its laceration, although not to a sufficient extent to impede the operation; during which she lost but little blood.

She was immediately conveyed to bed, and the house surgeon was instructed to be constantly with her, that he might be ready to act in case of hemorrhagy, but not to plug the vagina without absolute necessity. Four or five pallets of blood were voided in about twelve hours. She had nausea, vomiting, syncope, with griping pains, accompanied by frequent desire to evacuate both urine and feces. The hemorrhagy was not such as to require the plugging of the canal.

On the twenty-second day after the operation, I saw her in a most satisfactory state, and no doubt of her recovery existed.

Case II.—A female, aged about twenty-one, had been treated, for the space of two years, for an extensive ulceration and slight enlargement of the neck of the uterus. Having derived no benefit, she determined on submitting to the amputation of the part. Her state of body was not favorable for it. She had just recovered from a gastro-enteritis, by which she had been emaciated and enfeebled; but a fear that the progress of the disease might render the operation impracticable, made her anxious to have it speedily performed.

On the 8th of August it was performed by M. Lisfranc, in the Maison de Santé de la Rue de Valois. The relaxed state of the ligaments permitted the descent of the uterus low down, and the excision was made as before described. Slight hemorrhagy took place during the day, but no bad symptoms followed.

On the 18th, the wound was examined by means of the speculum, and found clean and healthy. Injections of chloruret of lime were used for the purpose of deterging the ulcer, and of hastening cicatrization.

On the 7th of September, slight granulations remained, which were touched by the liquid protonitrate of mercury.

15th.—Cure complete, and patient had recovered a healthy appearance.

---

*Arabian Mode of curing Fractured Limbs.*—The Orientals will never consent to have a limb cut off. Their practice is to lay the limb on an oiled mat after reducing the bones, and then enclose it in a case of gypsum, or plaster of Paris; an operation they perform much in the same way as is practised by statuaries to take a cast of a limb. They first pour the plaster of Paris under the limb, until it rises to such a height as to touch the whole lower surface, filling up all inequalities, so as to form a sort of bed; placing at the same time a few hollow reeds, at proper distances, and in such position as to serve to conduct away, through the plaster, any fluid that might collect in the gypsum case, from the wounds, &c. When this becomes firm, which it does in a very short time, the limb is next covered with the same plaster of Paris, so as to enclose it completely, and, on hardening, to form a light case, or plaster boot, to keep the parts in as natural a position as possible. They next make a sort of furrow, or channel, in the soft plaster, on the upper surface, to receive such vulnerary fluids, during the treatment, as they think conducive to the cure, and which filter through the gypsum to humect the leg at pleasure. To render this upper shell more easily removed or changed during the cure, if necessary, to examine the state of the parts, &c. they make deep incisions into the soft plaster, both lengthwise and across, though not quite through, by means of which the upper case is removed without disarranging the limb. The firmness of the lower part, or bed, makes the removal of the whole boot practicable, should such a measure at any time be found expedient.

Our readers will remember that Baron LARREY has lately practised a somewhat similar mode of treating fractured limbs, with much success. The Baron does not make use of a case of plaster of Paris, but surrounds the limb with bandages which have beeu soaked in some glutinous matter, which forms nearly as firm an envelope as the plaster of Paris.

### MIDWIFERY.

*Absorption by the Uterus.* (Extract of a letter from Dr. F. C. NAEGELE, Professor of Midwifery at the University of Heidelberg, to Dr. L. F. VON FRORIEP, of Weimar, Editor of a periodical publication entitled "Notizen aus dem Gebiethe der Natur und Heilkunde." Communicated by EDWARD RIGBY, M.D.)

During the year 1802, the following case occurred to my notice: A lady of high rank, in consequence, probably, of a somewhat fatiguing journey, from which she had just returned, was brought to bed between the twenty-fourth and twenty-sixth week of her pregnancy: the child lived several hours after birth ; little hemorrhage followed, but the placenta did not come away. The cord, which was very thin, had been torn off at its insertion, as far as could be judged from the length of it. The midwife, who was an experienced as well as a highly respectable person, informed me that it had occurred as she passed her finger along the cord to ascertain whether the afterbirth were already separated ; and assured me that she had not exerted too much force in endeavouring to extract it, in which account the by-standers also agreed.

The lady and her friends were under considerable alarm on account of the placenta not coming away; and the midwife, who suffered not less anxiety for her patient, scarcely quitted the bedside for the first nine days, and even passed the night in her room; so that the case was watched with the greatest attention.

The lochia, which were sparing and devoid of fetor, and with scarcely any coagula of blood, lasted only four days. A slight attack of fever was experienced twenty-four hours after delivery, unattended, however, with any pain of the abdomen. The breasts did not swell, the menstruation returned in eleven weeks, and in about three years after she bore a child at the full period of pregnancy.

In another case, in 1811, where abortion had occurred between the fourteenth and fifteenth week, from no assignable cause, and with scarcely any hemorrhage, and which I had an opportunity of observing with the greatest accuracy, the secundines did not come away; a febrile attack came on upon the third day, which soon disappeared; no local pain, no discharge from the parts of generation; the menses returned after nine weeks, and no traces of the placenta, &c. ever appeared.

An experienced accoucheur of this place (Dr. GÖTZENBERGER) has had the opportunity of attentively observing two cases of this kind, and assured me positively that he was perfectly convinced that no trace of the placenta had been detected, either in a solid or in a partly dissolved state.

January 19, 1828, I was sent for to attend a patient, aged twenty-four, the wife of a respectable farmer, living about ten or twelve miles from this place. She had been brought to bed of her second child the day before, at eleven o'clock in the morning, and the afterbirth had not yet come away. In the afternoon flooding had come on, which had been so considerable as to induce repeated fainting. Dr. Sigel, a physician of considerable experience, and M. Roth, an accoucheur, had been sent for from Ladenburg, and, on examination, had found retention of the placenta, in consequence of hourglass contraction of the uterus, which had prevented the hand being introduced for the purpose of separating and bringing it away. Tinct. Cinnamom. with a little laudanum, had been prescribed, and warm fomentations directed to be applied to the abdomen, to remove the spasmodic contraction. The flooding returned several times during the night and following day, and the discharge had begun to be very fetid.

I did not see the patient till thirty hours after delivery: I then found her very pale; the pulse quick, small, and somewhat excited; the uterus pretty firmly contracted, with no marks of hourglass contraction, but almost perfectly round; the discharge was extremely fetid, and I could feel a portion of the placenta within the os uteri. From the circumstances of the case, I considered the afterbirth as already separated; in which opinion the aforementioned medical gentlemen, and my friend, Dr. Rigby, (who had accompanied me,) agreed. Having deemed it necessary to remove it, I introduced my hand for this purpose, after experiencing considerable resistance from the contracted state of the uterus, and found the greater part of the placenta firmly attached to the uterus. This circumstance, combined with the obstinate and unruly behaviour of the patient, allowed me to separate and bring away scarcely two-thirds of the placenta: considerably more than one-third was left in the uterus, and the medical men present were convinced of the fact. The hemorrhage did not return. Injections of Infus. Fol. Salv. off. were repeatedly thrown up during the night and following day, but brought away scarcely any coagula of blood.

A smart attack of fever followed in about four-and-twenty hours after the operation, with violent headach, strong and full pulse, and considerable increase of temperature; the abdomen was not painful, even upon pressure; the breasts remained flaccid, although the child had been repeatedly applied by my order; and there were no signs of lochia. Almond emulsion, with nitrate of potass, and cooling drinks, were ordered; the bowels were evacuated by clysters, and an infusion of camomile flowers every now and then injected per vaginam. On the third day, however, the breasts began to swell, and a secretion of milk followed, but the child, which was weakly, refused the breast. The fever abated, and the milk again disappeared.

She enjoyed perfectly good health till the 27th of January, when the left eye was attacked with inflammation, which, in spite of all attempts to check it, rose to such a degree of intensity as, in a few days, to induce opacity of the lens and vitreous humor, with loss of sight in the organ. The menses returned in thirteen weeks after her delivery, in the usual quantity and duration; and at the present time, with the exception of blindness in one eye, she is in perfect health.

This case has been observed with the greatest attention, and the combination of circumstances that may be considered as having caused the ophthalmy, the obstruction to the secretion of milk, and suppression of the lochia, still further induced me to pay particular attention to the nature of the discharge.

As this circumstance interested me considerably, I have endeavoured of late years to excite the attention of several of my professional friends, and at various times have received accounts from them confirming the truth of my observations, both in cases of premature labour, where the placenta had been retained, as also of labour at the full term of pregnancy, where large portions of it had remained attached, where no traces of it in either in a solid or half-dissolved form had come away, and this had occurred without any injurious consequences.

My friend Professor SEBASTIAN, of this place, having lately returned from a journey to Holland, has communicated to me a most interesting case, which he received from the mouth of Dr. G. SALMON, of Leyden, where, after labour at the full period of pregnancy, the whole placenta had been absorbed, and the case terminated successfully. Dr. Salmon requested him to ask me if a case of this kind had ever come under my notice.

I am far from denying the liability to deception in cases of this sort, and am well aware how extremely difficult it is to form a correct opinion upon them. A comparison of this with processes of a similar nature, more especially with those that are observed to take place in cases of extra-uterine pregnancy, and also in animals, and a more elaborate discussion of the subject in a practical point of view, which has engaged my attention for some time, has made me very anxious to avail myself of the experience of others who may enjoy more extensive means for observation than myself. I have therefore taken the liberty of sending you this short and cursory communication, with the request that you will do me the favor of inserting it in your valuable Journal.—*Medical Gazette.*

---

*Double Uterus; Double Conception.*—Dr. GEISS, during his attendance on a lying-in patient, observed that the pains were entirely limited to the right side of the uterus, and that this side was elevated as high as the thorax; the left only extending to the umbilical region. The external organs of generation and os uteri were perfectly formed; and, on examination, the shoulder of a fœtus behind the membranes was distinctly felt. Immediately after the birth of the child, the right side of the abdomen diminished in volume; the left remained in *statu quo*. In about an hour after, labour pains again returned, and, on examination, Dr. G. discovered, beyond the orifice of the uterus, a membrane, distended by fluid, projecting through an annular opening in the left side of the uterus. The umbilical cord of the infant born passed to the upper part of a cavity similar to the uterus. On further examination, the abdomen of a child was distinctly felt at the opening above mentioned. Turning in this instance, as in the first, was necessary, which was not accomplished without difficulty. As the labour was not entirely finished, Dr. G. introduced his hand into the uterus, and thus convinced himself that this organ was double. The placenta in the right uterus was first thrown off, and the uterus contracted vigorously; but in the detachment of the second placenta, from the left uterus, the contractions were feeble, and the woman lost much blood: she, however, ultimately recovered.

Two years before, this woman had been delivered of one infant only, after a very difficult labour.—*Rust's Magazine*, vol. xx.

### NATURAL HISTORY.

*Lime* is one of the most universally diffused bodies in nature; and it would not be difficult to suppose that enough of this might be afforded by the aliment for ordinary purposes; but then there are some animals, as the testaceous and crustaceous, in which so large a quantity is requisite, as to make it impossible to consider the supply as depending entirely on the food taken in. Some accurate observations have been made as to the quantity of calcareous matter produced in eggs in a certain time, by a hen fed in a known way; and it has been satisfactorily ascertained that more calcareous matter was elicited than could be accounted for by that which was received as aliment. Here, therefore, there was either a generation of such calcareous matter by the powers of the system, or this substance must be a compound body, formed by some decompositions or new combinations of substances, whose chemical nature and mode of combination have not been sufficiently understood.

Similar difficulties have occurred in the vegetable kingdom; for, in answer to a prize question proposed by the Berlin Academy, to determine the constituents of the different kinds of corn, and to ascertain whether their earthy part is formed by the process of vegetation, it was at length discovered by SCHRADER, a Prussian, that seeds will grow, and produce corn yielding as much or more earthy matter than the original seed, when removed from all contact of earth, and watered merely with distilled water. The experiment was made on seed planted in sulphur, placed in a garden, at a distance from all dust, in a box to which the light and air had a free access, but from which all dust and rain were carefully excluded.

In confirmation of the same extraordinary circumstance, SAUSSURE found that plants growing in a calcareous soil, which contained little or no silica or flint, will nevertheless yield a considerable portion of that substance; and other chemists have discovered, in the ashes of some descriptions of pines, more than sixty-five per cent. of lime, when no traces of this substance could be found in the soil.

All these circumstances, therefore, discover that the powers of chemistry are inadequate to detect the processes which are continually carried on in the animal as well as the vegetable economy, for supporting life and promoting growth.—*Conversations on Animal Economy.*

---

*Indications of Wholesomeness in Mushrooms.*—Whenever a fungus is pleasant in flavor and odour, it may be considered wholesome: if, on the contrary, it have an offensive smell, a bitter, astringent, or styptic taste, or even if it leave an unpleasant flavor in the mouth, it should not be considered fit for food. The colour, figure, and texture of these vegetables do not afford any characters on which we can safely rely; yet it may be remarked that, in colour, the pure yellow, gold colour, bluish pale, dark or lustre brown, wine-red, or the violet, belong to many that are esculent; whilst the pale or sulphur yellow, bright or blood red, and the greenish, belong to few but the poisonous. The safe kinds have most frequently a compact brittle texture; the flesh is white; they grow more readily in open places, such as dry pastures and waste lands, than in places humid or shaded by wood. In general, those should be suspected which grow in caverns and subterraneous passages, on animal matter undergoing putrefaction, as well as those whose flesh is soft or watery.—*Quarterly Journal of Science.*

*Method of preserving Seeds fit for Vegetation.*—Fill an old cask half full of earth, put the seeds as near as possible to the middle of the cask, then fill the latter entirely with moist earth, pressing it down, and finally closing the cask so that neither air nor water may enter it. Keep it from contact of seawater. In this manner seeds may be brought from the East Indies or New Holland, in a state of perfect preservation and fit to vegetate.—*Gardener's Mag.*

---

*Preparation of Grain and Seeds by Chlorine.*—M. Remoud has been convinced, by numerous trials, that grain of all kinds, maize, the seeds of cruciform plants, potatoes, &c. by treatment with chlorine, are very much increased in vegetative power, are sooner ripe, and produce a crop three or four times as great as that obtained under ordinary circumstances. His process is to steep the seed for twelve hours in river water, (never in well water,) then fourteen or fifteen drops of a strong solution of chlorine is to be added to each litre (two pints) of water, the whole well mixed, and the maceration of the seed continued for six hours longer, in the sunlight and under a bellglass; or, for want of a bellglass, under a cover made with oiled paper. The seed is then to be separated from the liquid on a cloth, and, for the convenience of sowing, mixed with a sufficient quantity of cinders, sand, or dry earth. When sown, the water of maceration is to be poured over the ground. When possible, it is advantageous to water the ground once or twice, at long intervals, with water acidulated by muriatic acid, and in the same proportions as those mentioned. In addition to this process, the ground must be cultivated in the ordinary way.—*Courier de l'Ain. Bull. Univ.*

---

*Employment of Slates for hastening the Maturation of Fruits.*—A vine branch had been trained above the window of a house facing the south, according to custom in certain parts of France. Beneath this branch was a small slate roof, about three feet wide, serving to shelter a door. It was remarked that the grapes on this roof were ripe and black, while those on the rest of the branch were yet green. This effect, evidently due to the heat accumulated in the slates from the rays of the sun, has been advantageously employed in assisting the ripening of wallfruit.—M Bauchard, *Bull. Univ.*

---

*New Kind of Coffee.*—Endeavours have often been made in France to discover a substitute for foreign coffee. According to M. Pajot Descharmes, the seeds of the broom answer this purpose exceedingly well, according to the opinion of a person who has taken it for twelve years. Being moderately roasted, ground, and prepared in the manner of ordinary coffee, this person finds no difference between it and coffee. It is not the garden but the forest broom, the seeds of which are to be taken for this use. It appears that in that part of Holland bordering upon Germany this substance has been used instead of coffee for many years.—*Recueil Industrie.*

---

*Maturation of Wine.*—M. de St. Vincent, of Havre, states, from his own experience of long continuance, that when bottles containing wine are closed by tying a piece of parchment or bladder over their mouths, instead of using corks in the ordinary way, the wine acquires, in a few weeks only, those qualities which are only given by age in the ordinary way after many years.—*Nouveau Journal de Paris.*

### MISCELLANEOUS.

*Discovery of the Mode of Making the Diamond.*—At a meeting of the Academy of Sciences, on November 3d, M. GANAL stated that he employed phosphorus for the purpose of decomposing the carburet of sulphur, by which the carbon was set at liberty under the form of small crystals, having all the properties of the diamond, and possessing the power of cutting or scratching the hardest bodies. If sticks of phosphorus are introduced into a matrass containing carburet of sulphur, covered with a layer of water, as soon as the phosphorus comes in contact with the carburet, it dissolves as it would in water of 140° or 158° of Fahrenheit, and is precipitated to the bottom of the vessel. The mass, then, consists of three distinct layers: the upper part of pure water, the second of carburet of sulphur, the third of liquified phosphorus. If the liquor is agitated while in this state, so as to mix the different substances, it becomes milky and turbid, and, after remaining some time still, it separates anew, but apparently into two layers. The upper is formed by pure water, and the lower by phosphuret of sulphur. Between these layers is a very thin one of white powder, and which, when the matrass is held towards the rays of the sun, produces all the effects of a prism, and consequently seems to be formed by minute crystals.

The author, encouraged by this experiment, endeavoured to obtain more voluminous crystals, which he succeeded in doing by means of the following process: He introduced into a matrass, which was kept perfectly still, first eight ounces of water, then eight ounces both of carburet of sulphur and of phosphorus. As in the preceding experiment, the phosphorus first dissolved, and the three liquids took their stations in the vessel according to their specific gravities. After twenty-four hours, a very thin pellicle, consisting of a white powder intermixed with air bubbles and different centres of crystallization, was formed. After some days these pellicles gradually increased in thickness. The separation of the two lower liquids became less distinct, and after three months they seemed but one substance. The experiment having been left in action for another month, it became necessary to discover the mode of separating the crystallised substance from the phosphuret of sulphur, which was difficult on account of the inflammable nature of the substance. After various trials, more or less successful, the author determined to filter the whole through chamois leather, which he placed under a glass bell, renewing the air from time to time. At the end of a month the skin was washed and dried, when M. Ganal was enabled to examine the crystallised substance which remained on its surface. Exposed to the rays of the sun, it presented numerous crystals, reflecting all the colours of the rainbow: twenty among them were large enough to be raised with the point of the knife; three others were as large as a millet seed. The latter were submitted to the inspection of M. Champigny, director of the jewellery workshops of M. Petelot, and they appeared to him to be real diamonds.; M. Gay-Lusac stated that, to his knowledge, M. Ganal had been occupied in the same research for a period of eight years.

Five years since, in the month of January 1824, M. DELATOUR deposited with the French Academy of Sciences a paper, whose contents were then unknown, but have since proved to relate to the manufacture of the diamond, and contain, we presume, the results of the first essays of this gentleman. The method is still a secret, and said to be essentially different from that of M. Ganal just described.

On the 11th November, glass tubes were exhibited to the Academy, filled with diamond dust, or (to speak more accurately) carbon crystallised by art. The different specimens were not obtained by the same method. The chemical properties are the same, but in appearance and hardness they are strikingly different.

One of the tubes contains a very transparent small crystal, whose form is distinctly pyramidal. M. Delatour expects to present to the Academy crystals of four or five lines in diameter.

M. Arrago remarked on this occasion, that it would be easy to ascertain the nature of one of the crystals, as its "facettes" were sufficiently large to show the angle of prolongation. He stated also that a person of his acquaintance entertained a hope that the decomposition of carburet of sulphur by the voltaic pile would be successful. The defective conductibility of this substance had hitherto impeded the success of the experiment, but it is confidently expected that this difficulty will be overcome.—*From a Correspondent at Paris.*

